# Differential Expression of FosB Proteins and Potential Target Genes in Select Brain Regions of Addiction and Depression Patients

**DOI:** 10.1371/journal.pone.0160355

**Published:** 2016-08-05

**Authors:** Paula A. Gajewski, Gustavo Turecki, Alfred J. Robison

**Affiliations:** 1 Genetics Program, Michigan State University, East Lansing, Michigan, United States of America; 2 McGill Group for Suicide Studies, Douglas Mental Health University Institute and McGill University, Montréal, Québec, Canada; 3 Department of Physiology, Michigan State University, East Lansing, Michigan, United States of America; University of Colorado Boulder, UNITED STATES

## Abstract

Chronic exposure to stress or drugs of abuse has been linked to altered gene expression throughout the body, and changes in gene expression in discrete brain regions are thought to underlie many psychiatric diseases, including major depressive disorder and drug addiction. Preclinical models of these disorders have provided evidence for mechanisms of this altered gene expression, including transcription factors, but evidence supporting a role for these factors in human patients has been slow to emerge. The transcription factor ΔFosB is induced in the prefrontal cortex (PFC) and hippocampus (HPC) of rodents in response to stress or cocaine, and its expression in these regions is thought to regulate their “top down” control of reward circuitry, including the nucleus accumbens (NAc). Here, we use biochemistry to examine the expression of the *FosB* family of transcription factors and their potential gene targets in PFC and HPC postmortem samples from depressed patients and cocaine addicts. We demonstrate that ΔFosB and other FosB isoforms are downregulated in the HPC but not the PFC in the brains of both depressed and addicted individuals. Further, we show that potential ΔFosB transcriptional targets, including GluA2, are also downregulated in the HPC but not PFC of cocaine addicts. Thus, we provide the first evidence of *FosB* gene expression in human HPC and PFC in these psychiatric disorders, and in light of recent findings demonstrating the critical role of HPC ΔFosB in rodent models of learning and memory, these data suggest that reduced ΔFosB in HPC could potentially underlie cognitive deficits accompanying chronic cocaine abuse or depression.

## Introduction

The molecular and circuit-level mechanisms of psychiatric diseases such as depression and addiction are not fully understood, and this knowledge is crucial for rational development of new and better treatments. Alterations in gene expression in the nucleus accumbens (NAc) and the brain regions that exert top-down control over NAc function, like prefrontal cortex (PFC) and hippocampus (HPC), have been implicated in the pathogenesis of addiction and depression by many studies in both model organisms and in post-mortem human brain [1–5]. Many current treatments for depression operate through chronic enhancement of serotonergic and/or dopaminergic signaling, and virtually all drugs of abuse affect dopamine signaling in NAc. Moreover, addiction and depression are highly comorbid, with nearly one third of patients with major depressive disorder also having substance use disorders and comorbidity yielding higher risk of suicide and greater social and personal impairment [6, 7]. Taken together, these data suggest that chronic maladaptations in the mesolimbic dopamine circuit and connected structures may underlie both addiction and depression, and that changes in gene expression are likely to play a crucial role in these maladaptations.

As both depression and addiction develop over time and may be linked to chronic exposure to stress and/or drugs of abuse [8, 9], and because typical antidepressants that target serotonergic and dopaminergic signaling require weeks of treatment to be effective [10], it seems likely that the pathogenesis of these diseases and the mechanisms of their treatment may be linked to *long-term* changes in gene expression. Such changes could result from epigenetic modifications of gene structure, and indeed evidence is mounting for a key role for DNA methylation and histone modifications in both addiction and depression [11–14]. However, this does not rule out a potential role for transcription factors in these processes, particularly stable transcription factors induced by chronic neuronal activation. One such transcription factor is ΔFosB [1, 15, 16], a splice variant produced from the *FosB* gene. Unlike the full-length FosB protein, ΔFosB is remarkably stable in comparison to other immediate early gene products (half-life of up to 8 days in the brain [17]), primarily due to the truncation of two degron domains in the c-terminus [18], as well as a stabilizing phosphorylation at Ser27 [19, 20]. ΔFosB is induced throughout the rodent brain, including the NAc and related structures, by stress [21–23], antidepressants [22], and drugs of abuse [24]. Furthermore, rodent models implicate ΔFosB expression in NAc in both addiction [20, 25] and depression [26, 27], and recent studies suggest a role for ΔFosB in these diseases in PFC [21] and HPC [28]. In the NAc, ΔFosB expression promotes increased psychomotor sensitization to and reward from psychostimulants in rodents [20, 25]. NAc ΔFosB also acts as a proresilience factor in the mouse chronic social defeat model of depression, and its expression there is required for antidepressant function [26]. In contrast, expression of ΔFosB in PFC promotes susceptibility to social defeat stress in mice [21], suggesting that ΔFosB plays very different roles in the reward circuit and the brain regions that innervate it. Finally, ΔFosB is induced in the mouse dorsal HPC by learning and its function there is required for normal spatial memory formation [28], providing a possible mechanism for the cognitive deficits often accompanying chronic drug exposure and/or depression [29–31].

As ΔFosB is a transcription factor, it is commonly presumed that it exerts its biological effects through modulation of the expression of select target genes, and many of those target genes have been implicated in depression and addiction. ΔFosB regulates the expression of multiple subunits of α-amino-3-hydroxy-5-methyl-4-isoxazolepropionic acid (AMPA)- and N-methyl-D-aspartate (NMDA)-type glutamate receptors [25, 26, 32], and these receptors have been directly implicated in addiction [33, 34], depression [35, 36], and antidepressant function [36, 37]. ΔFosB also regulates the expression of signaling molecules, like calcium/calmodulin-dependent protein kinase II α (CaMKIIα), which has been linked to many psychiatric disorders [38], and we have shown that this regulation of CaMKII expression in mice drives psychomotor sensitization to cocaine [20] and antidepressant function [27]. In addition, ΔFosB regulates the expression of cyclin-dependent kinase 5 (cdk5) [39], which is induced in striatum by psychostimulant exposure and stress [40–42] and regulates the psychomotor and motivational responses to cocaine [43]. Thus, there is strong evidence in rodent models that induction of ΔFosB in multiple brain regions by stress, antidepressants, and drugs of abuse may regulate behaviors related to depression and addiction by modulating the expression of select target genes in discreet brain regions.

Although preclinical models of addiction and depression have been quite fruitful, it is essential to support findings from animal models with evidence from human studies if we expect to translate potential molecular mechanisms into novel treatment options. We have previously demonstrated that ΔFosB is upregulated in the NAc of human cocaine addicts [20] and reduced in the NAc of depressed humans [26]. However, regulation of *FosB* gene product expression in HPC and PFC, critical regulators of NAc neuronal activation, has not previously been studied in human brain, nor has regulation of potential ΔFosB target gene expression. We therefore examined the expression of *FosB* gene products, as well as the expression of potential ΔFosB target genes, in the PFC and HPC of patients suffering from major depressive disorder or cocaine addiction.

## Materials and Methods

### Human Samples

**Fig 1:**
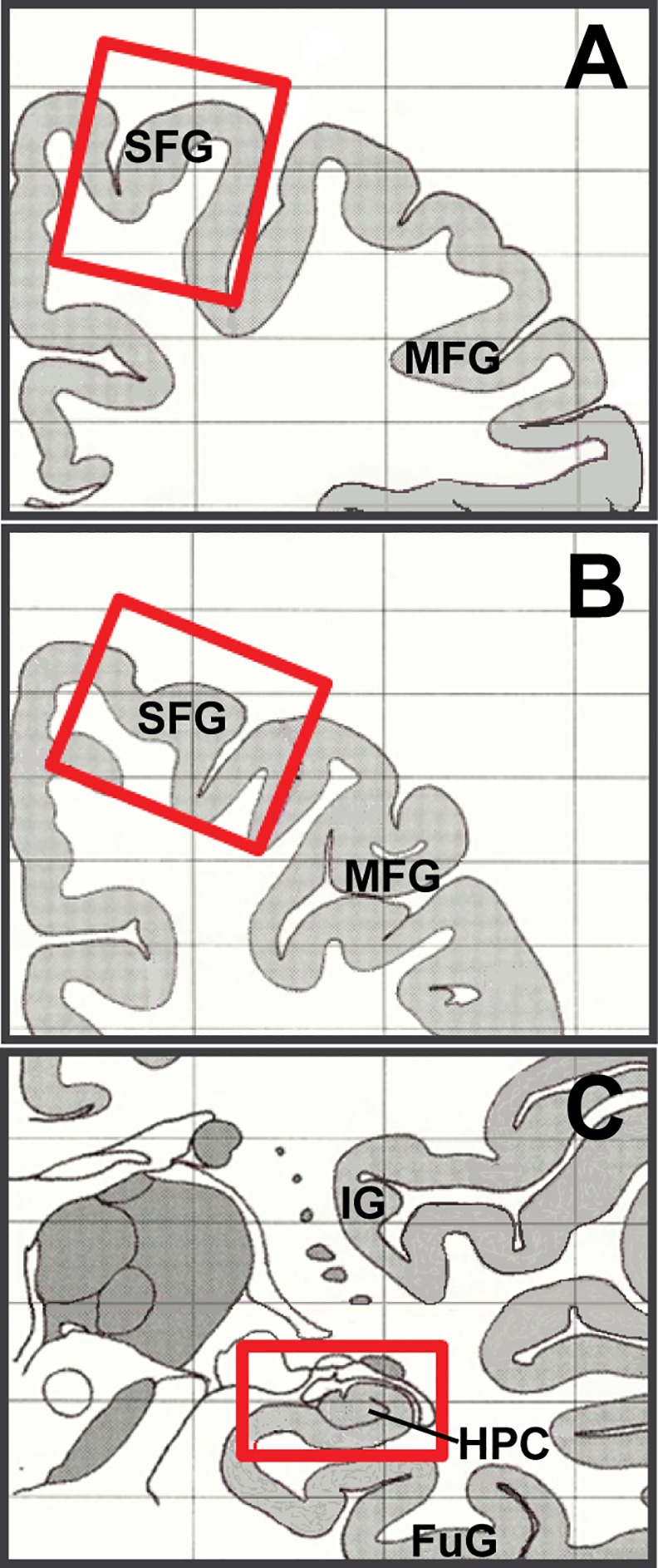
Diagram of dissection regions for human brain samples. Drawings represent anterior (A) and posterior (B) coronal sections of human brain used for dissection of PFC samples, and (C) HPC samples. Red boxes highlight areas of dissection. SFG: superior frontal gyrus; MFG: middle frontal gyrus; IG: insular gyrus; FuG: fusiform gyrus.

Post-mortem human brain tissues were obtained from the Douglas Bell-Canada Brain Bank (Douglas Mental Health University Institute, Montreal, Quebec, Canada). Substance use information regarding human cocaine addicts, depression patients, and matched controls can be found in Table 1. The preservation of tissue proceeded essentially as described [44]. Briefly, once extracted, the brain is placed on wet ice in a Styrofoam box and rushed to the Douglas Bell-Canada Brain Bank facilities. Hemispheres are immediately separated by a sagittal cut in the middle of the brain, brain stem, and cerebellum. Blood vessels, pineal gland, choroid plexus, half cerebellum, and half brain stem are typically dissected from the left hemisphere which is then cut coronally into 1 cm-thick slices before freezing. The latter half cerebellum is cut sagittally into 1cm-thick slices before freezing. Tissues are flash frozen in 2-methylbutane at -40°C for ~60 sec. All frozen tissues are kept separately in plastic bags at -80°C for long-term storage. Specific brain regions are dissected from frozen coronal slices on a stainless steel plate with dry ice all around to control the temperature of the environment. PFC samples come from Brodmann area 8/9, and HPC samples are taken from center mass of the hippocampal formation (Fig 1).

**Table 1:** Substance dependence, toxicology, and use of antidepressant medication in human cocaine addicts, depression patients, and matched control groups.

Group (number of subjects)	Additional Substance Dependence	Toxicology at Death (Drugs of Abuse)	Psychiatric Medication (Previous 3 months)
**Control (18)**	**• Alcohol (1/18)**	**Total = 2/18** • **Opioid (1/18)** • **Ethanol (1/18)**	**None**
**Cocaine Dependent (19)**	**Total = 15/19** • **Alcohol (11/19)** • **Cannabis (3/19)** • **Opioid (1/19)** • **Sedative (1/19)**	**Total = 14/19** • **Cocaine (11/19)** • **Opioid (2/19)** • **Ethanol (8/19)**	**Total = 6/19** • **SSRI/SNRI (3/19)** • **Benzodiazepine (5/19)** • **Classic Antidepressant (2/19)** • **Antipsychotic (2/19)**
**Control (11)**	**None**	**Ethanol (1/11)**	**None**
**Depressed Non-Medicated (14)**	• **Alcohol (5/14)**	**Total = 4/14** • **Opioid (1/14)** • **Ethanol (4/14)**	• **Benzodiazepine (2/14)**
**Depressed Medicated (13)**	• **Alcohol (1/13)**	**Total = 2/13** • **Opioid (1/13)** • **Ethanol (1/13)**	**Total = 13/13** • **SSRI/SNRI (9/13)** • **Benzodiazepine (6/13)** • **Classic Antidepressant (5/13)** • **Tricyclic Antidepressant (1/13)** • **Antipsychotic (3/13)**

Parentheses show number of patients with given condition out of number of total patients in the group. Note that some patients abused multiple additional substances or used multiple medications, thus the total number in a group with an additional condition is given as “Total.” SSRI: selective serotonin reuptake inhibitor; SSNI: selective norepinephrine reuptake inhibitor.

### Mouse Samples

**Fig 2:**
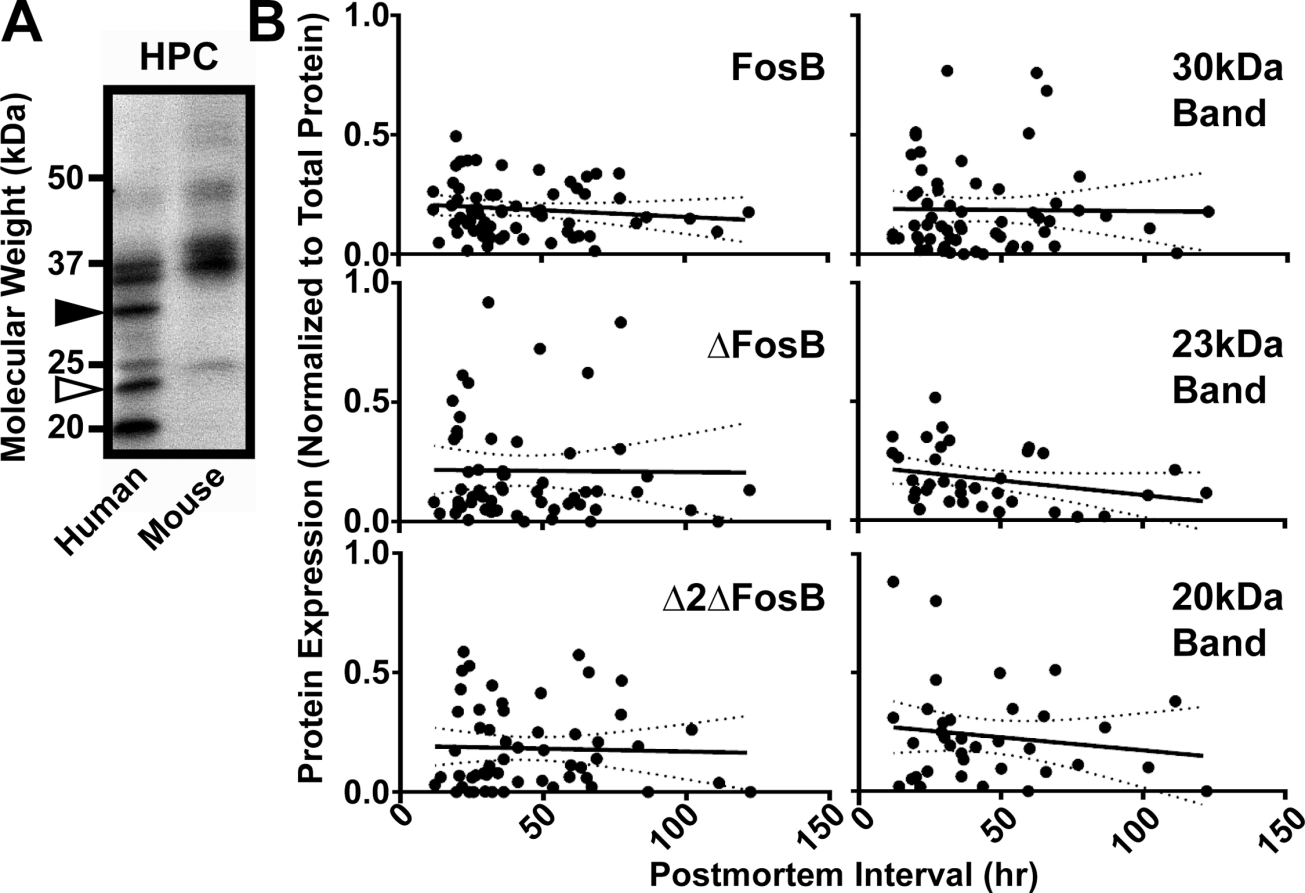
Comparison of human and mouse FosB proteins. (A) Western blot of hippocampal proteins with FosB antibody reveals multiple additional bands in typical human cocaine addict HPC sample compared to a chronic cocaine-treated (15 mg/kg for 7 days) mouse HPC. Novel bands are apparent at 20 kDa, 23 kDa (white arrow), and 30 kDa (black arrow). (B) Correlation and linear regression plots of protein expression for each band in the human samples with the postmortem interval (time between death and brain freezing) for each human sample. Dotted lines represent 95% confidence interval; no linear regression slope differed significantly from 0.

**Fig 3:**
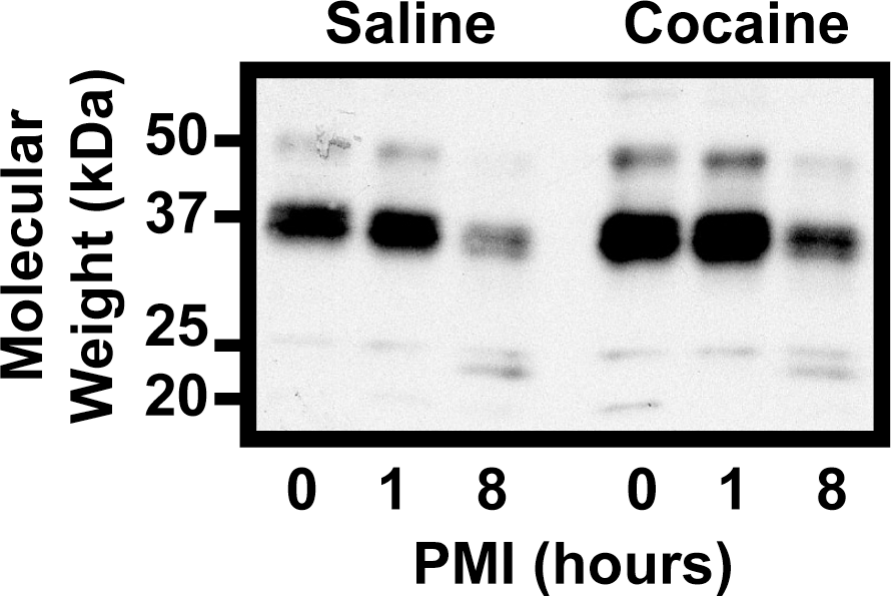
Expression of FosB proteins in mouse HPC after extended postmortem intervals. The brains of mice given an acute injection of cocaine (15 mg/kg i.p.) were left *in situ* for 0, 1, or 8 hrs after sacrifice before harvesting HPC. Western blot reveals buildup of a 23 kDa band in the 8 hr animals, but does not show other bands found in human HPC samples.

The study followed guidelines described in the *Guide for the Care and Use of Laboratory Animals*, eighth edition (Institute of Laboratory Animal Resources, 2011). Before any testing, all experimental procedures were approved by the Institutional Animal Care and Use Committee at Michigan State University. If any animal displays lack of grooming, infection, severe weight loss, or immobility, the animal is euthanized. No animals required such euthanization prior to experimental endpoint in the current study. After arrival to the facility, 7 week old C57BL/6 male mice (The Jackson Laboratory, Bar Harbor, ME, USA) were group housed at 4 per cage in a colony room set at a constant temperature (23°C) for at least 3 days before experimentation in a 12 h light/dark cycle with *ad libidum* food and water. Mice were given chronic (7 days) or acute (single injection) cocaine (15 mg/kg) or sterile saline (0.9% saline) via an intraperitoneal (i.p.) injection, and sacrificed by cervical dislocation one hour after the final injection. Tissue was harvested immediately (Fig 2) or at varying time points after sacrifice (Fig 3).

### Western Blotting

Mouse brains were extracted rapidly on ice and then sliced into 1 mm sections, and dorsal hippocampus was removed with a 12 gauge punch and immediately frozen on dry ice. Both human and mouse samples were homogenized by light sonication in modified RIPA buffer (10 mM Tris base, 150 mM sodium chloride, 1 mM EDTA, 0.1% sodium dodecyl sulfate, 1% Triton X-100, 1% sodium deoxycholate, pH 7.4, protease and phosphatase inhibitors [Sigma Aldrich]). Concentration was measured using DC Protein Assay (BioRad) and gel samples were normalized for total protein. Proteins were separated on 4–15% polyacrylamaide gradient gels (Criterion System, BioRad), and Western blotting was performed using chemiluminescence (SuperSignal West Dura, Thermo Scientific). Total protein was assayed using Swift Membrane Stain (G Biosciences) and proteins were quantified using ImageJ software (NIH). Primary antibodies were used to detect FosB isoforms (5G4; 1:500; Cell signaling, 2251), GluA2/3 (1:1,000; Millipore, 07–598), CaMKIIα (1:1,000; Millipore, 05–532), cdk5 (1:1,000; Santa cruz, sc-173), GAPDH (1:20,000; Cell Signaling, 21185).

### Statistics

All statistical analyses were performed using the Prism 6 software package (GraphPad). Linear regression analysis was used to determine whether the expression of *FosB* gene products was correlated with postmortem interval. The slope of each linear regression line was tested for significant difference from zero. Student’s t-tests were used for all pair-wise comparisons between control and cocaine addicted individuals (indicated in Results where t value is given). One-way ANOVAs were used for all multiple comparisons between controls, depressed individuals with antidepressants on board, or depressed individuals with no antidepressants (indicated in Results where F value is given). The one-way ANOVAs were followed by Tukey *post hoc* test. *P* < 0.05 was considered significant.

## Results

Our recent studies indicate that the three major products of the *FosB* gene in brain, full-length FosB (~50 kDa), ΔFosB (~35–37 kDa), and Δ2ΔFosB (~25 kDa), are differentially induced in mouse brain reward-associated regions in response to stress and antidepressant treatment [22], and other Fos-related antigens likely produced by the *FosB* gene have also been observed in mouse brain [45–47]. Therefore, we first sought to determine whether human brain expresses a pattern of *FosB* gene products similar to that found in mouse brain. We compared a typical HPC sample from a human cocaine addict (Table 2) to HPC from a mouse given chronic cocaine (15 mg/kg, i.p. for 7 days). All three major *FosB* gene products were found in both mouse and human brain tissue, but additional bands were observed in the human sample compared to mouse (Fig 2A). Most prominently, bands at ~30 kDa, ~23 kDa, and ~20 kDa appeared in human samples but were not observed in mouse samples. We postulated that these bands may represent proteolytic products resulting from degradation of FosB or ΔFosB due to the extended postmortem interval (PMI) in our human samples (Table 2). However, no correlation was found between the intensity of these novel bands and PMI (Fig 2B), or between PMI and the major gene products, FosB, ΔFosB, and Δ2ΔFosB (Fig 2B), i.e., none of the regression lines had a slope significantly different from zero. Thus, these novel bands may not be proteolytic degradation products resulting from prolonged time between death and tissue freezing.

**Table 2:** Demographics of human cocaine addicts, depression patients, and matched control groups.

Group (number of subjects)	Age	% Male	Brain pH	Postmortem Interval (h)
**Control (18)**	**33.05 ± 3.193**	**95%**	**6.569 ± 0.062**	**36.08 ± 4.515**
**Cocaine Dependent (19)**	**39.80 ± 2.153 p = 0.11**	**95%**	**6.546 ± 0.072 p = 0.48**	**42.78 ± 4.661 p = 0.31**
**Control (11)**	**41.58 ± 3.241**	**83%**	**6.508 ± 0.074**	**31.75 ± 5.899**
**Depressed Non-Medicated (14)**	**48.14 ± 3.061**	**71%**	**6.721 ± 0.055**	**39.04 ± 6.478**
**Depressed Medicated (13)**	**45.75 ± 2.713 p = 0.33**	**69%**	**6.671 ± 0.083 p = 0.14**	**40.66 ± 7.357 p = 0.65**

All values are mean +/- standard error. P values are calculated using two-tailed student’s t-test for cocaine samples and one-way ANOVA for depression samples. 100% of participants were Caucasian.

To further investigate this, we gave mice a single injection of cocaine (15 mg/kg, i.p.) or saline and sacrificed them by cervical dislocation one hour later. The brains were then left *in situ* for zero, one, or eight hours before samples were taken. We noted some degradation products (Fig 3), the most prominent being ~23 kDa, but the resulting pattern did not mimic that seen in human HPC samples. Taken together, these data indicate that there are additional Fos-related antigens in human brain that may represent novel *FosB* gene products and are unlikely to be the result of proteolysis of FosB or ΔFosB.

**Fig 4:**
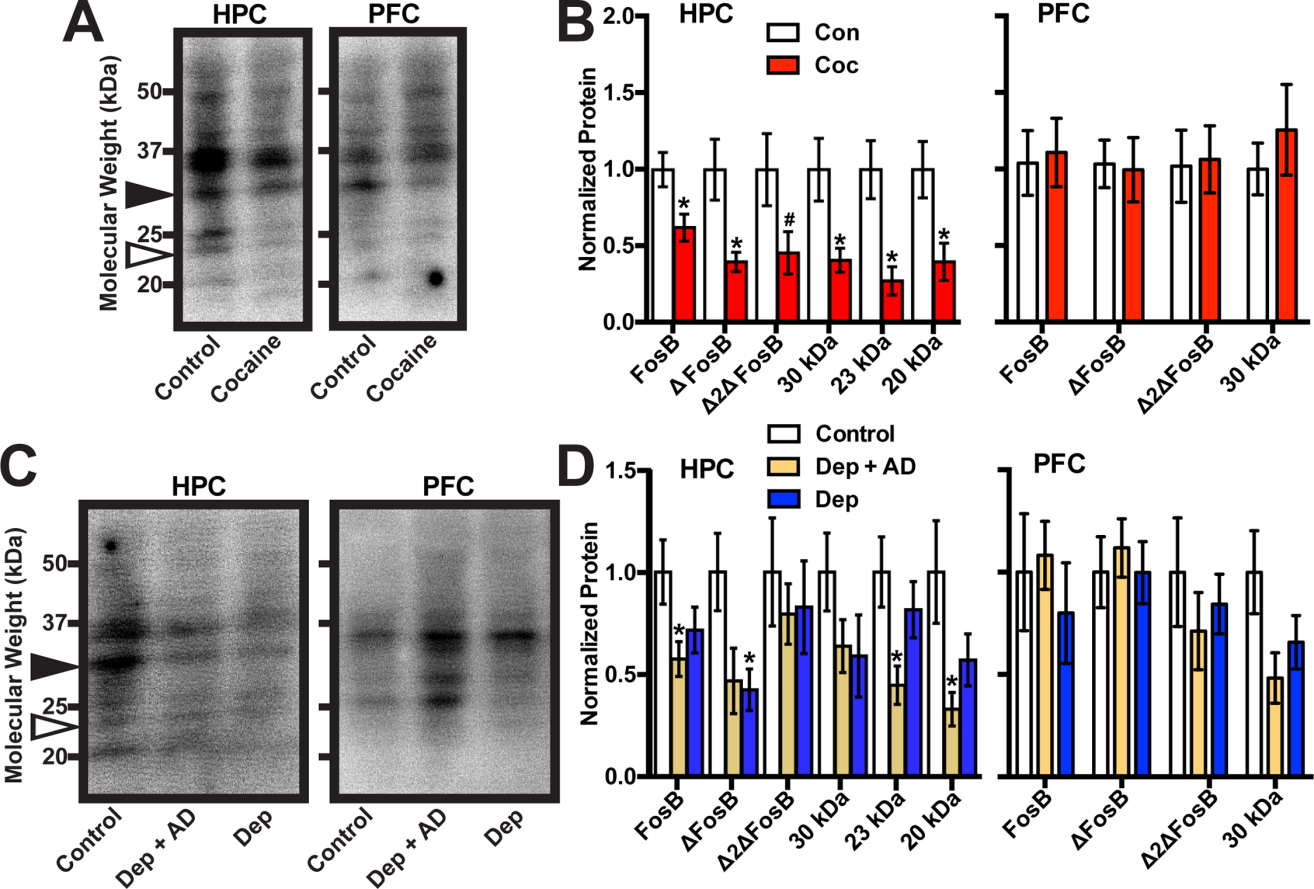
Expression of FosB proteins in HPC and PFC of human cocaine addiction and depression patients. (A) Western blot of FosB proteins from the HPC and PFC of human cocaine addicts (Coc) and controls (Con). (B) Quantitation reveals a cocaine-dependent decrease in many FosB proteins in the HPC but not PFC (*:p<0.05, #:p = 0.05). (C) Western blot of FosB proteins from the HPC and PFC of human depression patients off (Dep) or on antidepressants (Dep + AD) and controls (Con). (D) Quantitation reveals a depression-dependent decrease in some FosB proteins in the HPC but not PFC (*:p<0.05). Error bars indicate mean +/- SEM.

We next sought to determine whether cocaine dependence, untreated depression, or depression coupled with exposure to antidepressant medication are associated with changes in *FosB* gene products in human HPC or PFC. Patients and control subjects were chosen such that there were no significant differences in average age, gender, brain pH, or PMI (Table 1). In samples from cocaine dependent patients, Western blot revealed no differences in the expression of any FosB isoform in the PFC compared to controls (Fig 4A and 4B). However, we observed a marked decrease in the HPC of cocaine dependent individuals in full-length FosB (*t*(35) = 2.67, *p* = 0.012), ΔFosB (*t*(31) = 2.81, *p* = 0.009), as well as in all three novel bands, 30 kDa (*t*(34) = 2.71, *p* = 0.011), 23 kDa (*t*(15) = 2.7, *p* = 0.016), and 20 kDa (*t*(13) = 2.43, *p* = 0.031), and a trend toward a decrease in Δ2ΔFosB (*t*(29) = 2.03, *p* = 0.052). Similarly, in samples from patients suffering from depression, there were no differences in the expression of any FosB isoform in the PFC, while the HPC showed decreases in full-length FosB (F(2,35) = 1.98, p = 0.048) and ΔFosB (F(2,30) = 1.38, p = 0.027), as well as in the 23 kDa band (F(2,21) = 2.05, p = 0.022) and the 20 kDa band (F(2,18) = 0.97, p = 0.028) (Fig 4C and 4D). These data suggest that *FosB* gene expression in HPC is reduced in multiple psychiatric conditions while PFC expression is unaffected.

**Fig 5:**
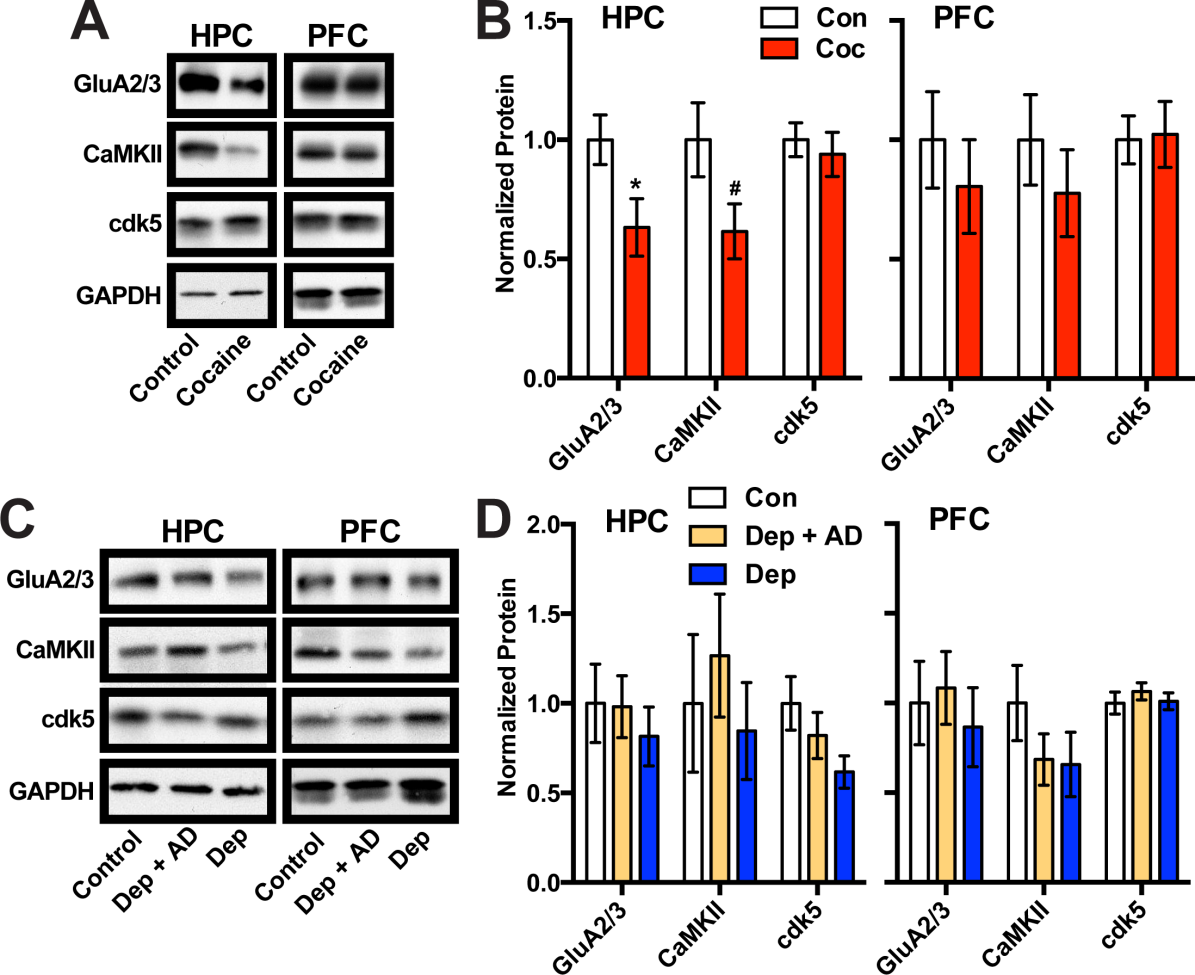
Expression of possible ΔFosB gene target proteins in HPC and PFC of human cocaine addiction and depression patients. (A) Western blot of potential ΔFosB gene target proteins from the HPC and PFC of human cocaine abusers (Coc) and controls (Con). (B) Quantitation reveals a cocaine-dependent decrease in all GluA2 and CaMKII in the HPC but not PFC (*:p<0.05, #:p = 0.05). (C) Western blot of potential ΔFosB gene target proteins from the HPC and PFC of human depression patients off (Dep) or on antidepressants (Dep + AD) and controls (Con). (D) Quantitation reveals no depression-dependent changes. Error bars indicate mean +/- SEM.

Direct evidence for gene targets of ΔFosB transcriptional regulation in HPC is scant, with only cyclin-dependent protein kinase 5 (cdk5) a confirmed target after electroconvulsive stimulation in mice [39]. However, many other genes are known targets for ΔFosB transcriptional regulation in other brain regions, particularly in NAc. These include a number of genes essential for hippocampal cell function and synaptic plasticity, such as GluA2 [48] and CaMKII [20]. Therefore, we used Western blot to assess the levels of potential gene targets of ΔFosB in HPC and PFC of cocaine dependent and depressed patients. We found no significant differences in the protein levels of the candidate target genes in the PFC of cocaine dependent individuals, while the HPC showed a significant decrease in GluA2 (t(34) = 2.31, p = 0.027) and a strong trend toward decrease in CaMKII levels (t(35) = 1.99, p = 0.053) expression, while cdk5 remained unchanged (Fig 5A and 5B). In the PFC and HPC of depressed patients there were no changes in expression of the ΔFosB target genes (Fig 5C and 5D). These data suggest that ΔFosB may be regulating the expression of potential target genes in human HPC, and this regulation may be brain region and disease specific.

## Discussion

Here, we present the first compilation of *FosB* gene product and ΔFosB-target protein analysis in the hippocampus and prefrontal cortex of cocaine addicts and depressed patients. These brain regions are known to play key roles in the pathophysiology of these diseases, and the use of human post-mortem samples allows us to: 1) determine whether the molecular alterations found in well-studied rodent models of these diseases are recapitulated in humans; 2) identify novel pathways for study in rodent models for potential therapeutic intervention. Our analyses focused on the expression of *FosB* gene products, as their expression in these regions has been suggested to play a role in depression and is induced by cocaine exposure in rodent models [21, 22, 24]. When initially examining the FosB protein levels in our human samples, it was clear that our FosB antibody detected more bands than have previously been reported in rodent brain samples by our group and many others [1, 22]. Because human brains are frozen hours after death while mouse samples are removed and frozen within two minutes of sacrifice, we left mouse brains *in situ* after sacrifice for up to eight hours to determine whether similar bands would emerge. However, because we did not observe the same pattern of FosB proteins found in the human samples, and because we also found no correlation between the length of PMI and the levels of the various bands in human samples, we concluded that many of the bands in the human brain samples are unlikely to be the result of proteolytic degradation of larger FosB isoforms. Although we cannot rule out differences in the proteolytic machinery between species, we would suggest that some of the human bands may result from differential splicing of the FosB mRNA, and future studies from our group will address this question.

Previous results from rodent studies have found an increase in FosB isoforms in HPC and PFC after chronic cocaine [24]. However, from our cohort of cocaine dependent individuals we found a decrease in all FosB isoforms in HPC, with no change in PFC compared to control individuals. We believe this may be due to the inherent differences between rodent studies and cases of human addiction. Studies of cocaine addiction only last for a small fraction of the rodent’s life, and no ΔFosB induction studies to date have gone beyond 14 days of continuous cocaine exposure [1, 20]. Human cocaine users can be addicts for much longer time periods, which may induce homeostatic effects causing the *FosB* gene to be repressed in HPC. Moreover, many studies have demonstrated that long-term addiction to psychostimulants is accompanied by reduced cognitive function [9, 49]. Our recent work demonstrates that HPC ΔFosB plays a critical role in learning [28], and thus the decrease in HPC *FosB* gene expression in cocaine addicts shown here may represent a mechanism for cognitive decline in psychostimulant addiction. With decreased expression of the *FosB* gene in HPC, we also observed a decrease in the protein levels of candidate ΔFosB target genes GluA2 and CaMKII, and both of these molecules are also critical for HPC function and learning [50] and have been previously linked to addiction [38, 51].

In the HPC of depressed patients, we observed a decrease in multiple FosB proteins, depending on whether patients were taking antidepressants. This may indicate that antidepressants have differential effects on the splicing or stability of *FosB* gene products, though our previous studies in rodents did not reveal any such differences [22]. However, there were no differences in the expression of potential target genes in either HPC or PFC of these patients. Although major depression is often accompanied by cognitive problems [52], it is likely that HPC ΔFosB is not the only factor altered in response to depression. While the cocaine addicts showed changes in HPC ΔFosB and in target gene expression, depression may be leading to different compensatory mechanisms that prevent reduction in GluA2 or CaMKII expression. Thus, future studies will elucidate whether changes in HPC gene expression in depression and addiction arise from similar mechanisms.

It is critical to note that the human populations used for this study lack the homogeneity of preclinical rodent or primate models. For instance, five of the depressed patients suffered from alcoholism, and two had opiates on board at time of death. Similarly, six of the cocaine-dependent individuals had used antidepressants in the three months prior to death. Although this is not surprising, since depression and addiction have a high level of comorbidity [6, 7], it does complicate interpretation of results. We do not observe a significant difference in any of our biochemical measures between cocaine-dependent subjects who had antidepressants on board and those that did not, nor do we observe differences between depressed patients who had substance dependence and those who did not (data not shown). However, this does rule out overlapping or synergistic effects of depression and addiction on our measures. On the contrary, as we observe similar decreases in HPC FosB isoform expression with depression and addiction, it is possible that reduction in HPC *FosB* gene expression is a common mechanism between the two conditions and may contribute to comorbidity. Investigation of this hypothesis will require much larger cohorts of human subjects and additional preclinical studies.

In conclusion, we find that multiple *FosB* gene products are downregulated in the HPC, but not the PFC, of humans suffering from addiction and depression. Although we cannot make an etiological connection between this phenomenon and the disease states, it is possible that decreases in HPC ΔFosB and/or other FosB isoforms may in part underlie the cognitive deficits associated with depression and addiction, or contribute to the comorbidity of these psychiatric disorders.
